# Status Dystonicus with Atypical Presentation in Developmentally Delay Child: A Case Report

**DOI:** 10.31729/jnma.7614

**Published:** 2022-08-31

**Authors:** Shailendra Kumar Yadav, Pratibha Yadav, Gyabina Maharjan, Sujata Dahal, Nirajan Khati

**Affiliations:** 1 Department of Pediatrics, Ishan Children and Women's Hospital, Basundhara, Kathmandu, Nepal; 2 Department of Pediatrics, The Second Affiliated Hospital of Kunming Medical University, Kunming, Yunnan, China; 3 Department of Pediatrics, Kirtipur Hospital, Kirtipur, Kathmandu, Nepal; 4 Department of Medicine, Patan Academy of Health Sciences, Lagankhel, Lalitpur, Nepal; 5 Department of Paediatrics, Vayodha Hospitals, Balkhu, Kathmandu, Nepal

**Keywords:** *aspiration*, *children*, *dystonia*, *epilepsy*, *pneumonia*

## Abstract

Status dystonicus is characterised by involuntary sustained or intermittent muscle contractions of muscles causing repetitive twisting movements, abnormal postures of the body, or both is a rare but life-threatening movement disorder. Early diagnosis and management of status dystonicus prevent serious complications. We report a 2 years old previously developmental delay diagnosed girl who presented with generalised contractions of the whole body. Tightening of limbs is aggravated by touching her backside which is a very unique feature. Dystonia is associated with severe sweating and was confused with a seizure event. The patient was treated with midazolam, clonidine, phenytoin, gabapentin, pyridoxine, baclofen, and trihexyphenidyl. She was admitted to the intensive care unit for monitoring. The patient partially recovered after 10 days of treatment.

## INTRODUCTION

Status dystonicus (SD) is a severe and life-threatening movement disorder in children and affects all age groups but is also seen in older children up to 60%.^1^ The mortality rate is 10%. The exact risk factors are unknown however pain, fever, and dehydration are the main trigger factors.^2^ Painful and recurrent spasms may lead to respiration and metabolic disturbances such as hyperpyrexia, dehydration, respiratory insufficiency, and acute renal failure secondary to rhabdomyolysis. Management includes supportive care, treating underlying causes, and anti-dystonic.^3^ Medically refractory cases are managed surgically. This type of case requires more caution. Here, we present a case of a SD in children.

## CASE REPORT

The 2 years old child was referred to our hospital for management of a seizure. Upon assessment, there was no family history of neurological disorders, and she did not use any regular medication before onset. She had one episode of seizures when she was one year old and was diagnosed with autism with developmental delay. She was able to perform all normal activities, 6 days before she developed sustained, painful, right laterocollis and sustained painful right wrist flexion , and hand contraction. After being brought to the hospital antiseizure medications including midazolam, phenytoin, and valproate were given. Even though the seizure was controlled for only two hours. Following that, she had more than 1 hour spastic generalized contraction of the whole body. The tightening of limbs is aggravated by touching her backside. Dystonia is associated with severe sweating. When the mother tries to feed her, contraction of limbs occurs along with bending of vertebrae within 2 minutes, which look like opisthotonus. When there were no dystonic features then she started to laugh and smack her lips. She was unable to eat, drink, or sleep due to bizarre neck positioning and pain. She had a recent upper respiratory infection but no other triggers. Due to her unresponsiveness to intravenous lorazepam, she was transferred to the pediatric intensive care unit (PICU). Sensation and coordination couldn't be assessed. Reflexes were brisk and symmetric.

**Figure 1:**
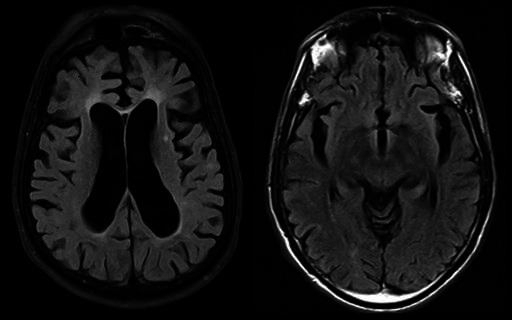
Non-contrast MRI brain showing atrophic changes in bilateral frontotemporal lobes.

The patient was awake and alert and in moderate distress. Her mental status was normal, and her speech was not clear. Chest X-ray showed right lower lobe consolidation. Magnetic Resonance Imaging (MRI) brain showed atrophic changes in bilateral frontotemporal lobes (Figure 1).

On subsequent pathology reports, all blood parameters, cerebrospinal fluid analysis (CSF) were normal. The exact causes of dystonia were unknown. She was given intravenous fluid with isotonic crystalloids. She was administered clonidine, valproate as well as midazolam. The above treatment plan was continued for 24 hours, with the understanding that if it was unsuccessful, endotracheal intubation and deep sedation would be performed. The patient was monitored for response and exhaustion signs. The current therapy was continued, and intubation was foregone. After additional 48 hours of clonidine, phenytoin, gabapentin, pyridoxine, baclofen, and trihexyphenidyl. She improved but due to neck muscle contraction, she was given food and fluids through a nasogastric tube. She was transferred to the general ward and continued all the anti-seizure medications. Even after the continuation of antiseizure medication, she had a spastic contraction of limbs which mostly exaggerated with feeding and touching her backside. After 10 days of general ward living, her condition was partially improved and was discharged with valproate, baclofen, gabapentin, and clonidine.

## DISCUSSION

SD is a rare movement disorder emergency associated with significant morbidity and life-threatening events that requires immediate and effective treatment. SD nowadays is currently an under-recognized and undertreated condition, partly due to the lack of a standard definition and because it can be the acute complicated course of both primary and secondary dystonias. Brain damage and genetic or environmental factors (e.g. fever and dehydration) are trigger factors. Even in approx 32.6% of cases precipitation factors remain unclear. Early diagnosis and management of SD prevent serious complications.^4^

All age groups are affected by SD, although the age groups of 5 and 16 are also affected up to 60% .^5^ The incidence of SD in all children aged <14 years admitted to a single centre with SD between 2014 and 2018 were studied. Results showed among twenty-four children 75% male were identified with SD. In new admission children <12 years of age, the annual incidence rate was 0.05 per 1000. The mean age at presentation was 6.3±3.6 years. The most common triggering factor was intercurrent illness/infection. Cerebral palsy was the most common. Other causes included complicated tubercular meningitis and mitochondrial disorders. Basal ganglia involvement. Due to respiratory muscles involvement causing respiratory problems. Total of 3 children died owing to refractory SD and its complications; the mortality rate is 10%.

The common movement disorders are chorea, dystonia, tremor, myoclonus, and parkinsonism in descending order of frequency. In this series of mainly previously well children with cryptogenic acute movement disorders, three groups are recognized: (1) Psychogenic disorders, typically >10 years of age, more likely to be female and to have tremor and myoclonus (2) Inflammatory or autoimmune disorders, including N-methyl-d-aspartate receptor encephalitis, opsoclonus-myoclonus, Sydenham chorea, systemic lupus erythematosus, acute necrotizing encephalopathy (which may be autosomal dominant), and other encephalitides and Non-inflammatory disorders, including drug-induced movement disorder, post-pump chorea, metabolic, e.g. glutaric aciduria, and vascular disease, e.g. moyamoya.^6^ Other important non-inflammatory movement disorders, which are mostly seen in symptomatic children with underlying etiologies such as trauma, severe cerebral palsy, epileptic encephalopathy, Down syndrome, cerebral palsy, and Rett syndrome, include dystonic posturing secondary to gastro-oesophageal reflux (Sandifer syndrome) and Paroxysmal Autonomic Instability with Dystonia (PAID) or autonomic 'storming'. RNA polymerase II is a multiprotein complex function that catalyzes gene transcription. Primary dystonia is due to TOR1A mutation and epileptic encephalopathies such as ARX and GNAO^1^ genetic variants and neurodegenerative disorders such as PANK^2^ which is characterised by neurological features like disabling, abnormal, involuntary movements whereas secondary dystonia develops due to environmental factors that cause injury to the brain. Exome sequencing detected biallelic putative disease-causing variants in MED27, encoding Mediator complex subunit 27. Patient phenotypes are highly homogeneous, including global developmental delay, intellectual disability, axial hypotonia with distal spasticity, dystonic movements, and cerebellar hypoplasia. Dystonic cerebral palsy is the most common acquired dystonias. The severely affected individuals have seizures and cataracts. Identification of multiple patients with biallelic MED27 variants supports the critical role of MED27 in normal human neural development, particularly for the cerebellum.^7^

The cerebellum has played a great role in controlling motor functions such as coordination, balance, posture, and skilled learning. cerebellar lesions are associated with a wide array of diseases including ataxia, dystonia, tremor, schizophrenia, dyslexia, and an autism spectrum disorder.^8^

The classical distinction is very hard to differentiate between isolated and combined dystonias since many genes have been shown to determine multiple dystonic presentations (e.g., ANO3, GNAL, ADCY5, and ATP1A3). In addition, a growing number of genes initially linked to other neurological phenotypes, such as developmental delay, epilepsy, or ataxia, are now recognized to cause prominent dystonia, occasionally in an isolated fashion (e.g., GNAO1, GNB1, SCN8A, RHOBTB2, and COQ8A). Emerging analyses showed biological convergence of genes linked to different dystonic phenotypes. monogenic dystonias have tremendously grown, and their clinical boundaries are becoming increasingly blurry.^9^

The delayed management of dystonia and early recognition of worsening dystonia may potentially facilitate intervention or prevent progression to SD. However, for established SD, the most immediate and effective modalities for abating life-threatening spasms, while dystonia-specific treatment takes effect. For children with idiopathic focal or generalised dystonia, carbidopa-levodopa can be used for both aetiology-specific treatments as well as symptomatic therapy. If not responded by levodopa then botulinum toxin injection for focal dystonia and generalised dystonia anticholinergic drug-like trihexyphenidyl or benztropine is used. However generalised dystonia is unresponsive to medical therapy then it is managed surgically (deep brain stimulation). High-dose baclofen is used for idiopathic generalised dystonia but it causes serious side effects, including seizures if sudden withdrawal.^10^ Deep brain stimulation (DBS) has emerged as an effective treatment for SD which is refractory to medical management, the children are affected by methylmalonic acidemia and suffer acute basal ganglia lesions, while the other carries a pathogenic mutation in the GNAO1 gene. DBS targets the subthalamic nucleus (STN) and globus pallidus internus (GPI). SD resolution within 8-19 days after surgery. Also, the recurrence rate is highly reduced. Gene therapy is also given in the case of inherited dystonia.^11^ The course and outcome of SD are highly variable; male gender and prevalent tonic phenotype have a poor prognosis.^12^

It provides us information about how to recognize children at high risk of SD and identify early the subtle clinical signs before the onset of an SD. Physicians need to consider epilepsy and seizure disorder in the differential diagnoses of any sudden onset focal neurological deficit. Delay in diagnosis may lead to multi-organ-dysfunction syndrome and death, otherwise curable disease. Close and prolonged follow-up should be performed for detecting recurrence and its complications.
